# Trait-like individual signatures dominate sleep EEG over insomnia-specific features

**DOI:** 10.1038/s41598-025-34509-y

**Published:** 2026-01-06

**Authors:** Markus Kyllönen, Roy Cox, Tommi Makkonen, Risto Halonen, Lauri Parkkonen, Emil Hein, Eus J. W. Van Someren, Anu-Katriina Pesonen

**Affiliations:** 1 SLEEPWELL Research Program, Faculty of Medicine, University of Helsinki, Haartmaninkatu 3, 00014 Helsinki, Finland; 2 Department of Sleep and Cognition, Netherlands Institute for Neuroscience, Royal Netherlands Academy of Arts and Sciences, Amsterdam, The Netherlands; 3 Department of Psychology, Faculty of Medicine, University of Helsinki, Helsinki, Finland; 4 Department of Neuroscience and Biomedical Engineering, Aalto University School of Science, Espoo, Finland; 5 Department of Psychiatry, Amsterdam Public Health Research Institute and Amsterdam Neuroscience Research Institute, Amsterdam UMC, Vrije Universiteit, Amsterdam, The Netherlands; 6 Department of Integrative Neurophysiology, Center for Neurogenomics and Cognitive Research (CNCR), Amsterdam Neuroscience, Vrije Universiteit Amsterdam, Amsterdam, The Netherlands

**Keywords:** Sleep, Insomnia, Polysomnography, EEG, Machine learning, Spectral analysis, Neurology, Neuroscience

## Abstract

Insomnia-specific features of sleep EEG activity have remained elusive, with existing findings being inconsistent and often weak in effect. Using machine learning, we analyzed two independent electroencephalogram (EEG) datasets spanning two nights (N_subjects/nights_=198/396), comprising individuals with insomnia disorder (ID) (mild to moderate/severe) and good sleeper controls (GSCs). The findings demonstrated that sleep EEG spectral features differentiated ID from GSC only when using identical participants for training and testing, indicating that model performance was driven by individual EEG signatures instead of ID-related patterns. Analyses with unsupervised learning, similarity matrices, and periodicity assessments further confirmed that brain activity during sleep is characterized by robust, individual-specific EEG signatures with trait-like stability over two nights. We also show that the individual sleep EEG signatures are driven by high frequency cortical activity, previously associated with cortical arousal during sleep. The results then demonstrate that high frequency cortical activity is not specific to ID, but the key to characterizing individual sleep EEG signatures. While ID may be characterized by EEG features beyond spectral power, our findings underscore the importance of a precision brain health framework that prioritizes deviations from an individual’s own neural baseline rather than relying solely on group-level comparisons.

**Supplementary Information:**

The online version contains supplementary material available at 10.1038/s41598-025-34509-y.

## Introduction

Despite the high prevalence of insomnia disorder (ID), evidence regarding its associated brain activity features during sleep remains controversial. A common discrepancy is observed between the subjective experience of insomnia and the fundamental and objective sleep measures derived from sleep electroencephalogram (EEG)^1,2^ recordings. The observed variability may stem from the variety of analytical approaches and targets and from the heterogeneity of the data and ID phenotypes^2,3^.

The prevailing model of ID points to somatic, cognitive and/or central nervous system (CNS) hyperarousal as the key feature of ID^4–9^. Hyperarousal during sleep may associate with more fragmented rapid-eye-movement (REM) and non-REM (NREM) sleep^2,8^, EEG signatures reflecting lighter sleep^10^, and with a greater sleep misperception linked to ID (perceived wakefulness while asleep)^8,11,12^. The biological mechanisms underlying sleep-related hyperarousal are suggested to associate with a locus coeruleus-noradrenaline (LC-NA) activity, providing a vigilance-promoting mechanism that renders sleep vulnerable to disruption through sustaining thalamocortical and autonomic sensory arousability^2,13,14^. It has been suggested that in insomnia, the LC is sensitive to - or receives more input from - the salience network and related circuits, even during REM sleep, when it should normally be sound asleep^8^. Restless REM sleep in specific, may alter synaptic plasticity in limbic circuits and interfere increasingly with the overnight resolution of distress^8,15^ leading to gradual accumulation of hyperarousal, and ultimately to chronic insomnia.

To date, the empirical evidence on increased high-frequency cortical activity observed during sleep in individuals with ID remains controversial. Since the first review, summarizing seven studies and showing an increased beta activity with ID^5^, the picture has become more varied, pointing to both increased cortical arousal^1, 16–21^ but also lack of differences across ID and GSC^22, 23, 24–26^. A meta-analysis^3^ summarized 24 studies (*N* = 977; 54% with ID) and indicated higher beta activity in absolute power spectral density (PSD) in ID compared to GSC, but only during NREM sleep. Since the meta-analysis, two large studies have produced mixed results on the topic^27,28^.

A question that has received less attention is whether the possibly deviating EEG spectra in individuals with insomnia fluctuate across nights^29^ or reflect stable, trait-like characteristic similar to that seen in the general population during rest^30,31^ and sleep^32–36^. Insomnia studies have not thoroughly considered these individual differences, as the studies primarily rely on average group comparisons based on one night.

Studies utilizing ML algorithms have reported high accuracy rates – often exceeding 90% – in detecting insomnia from EEG spectral activity^37–41^. However, the decision-making processes of these models remain largely non-transparent, making it unclear which factors most significantly influence their predictions. Furthermore, many of these studies rely on the same dataset (The Physionet CAP Sleep database^42,43^ for their analyses, which may present a confounding correlation between insomnia and age^44^.

To better capture the complexity of brain activity during sleep, a multidimensional analytical framework that accounts for parallel and interdependent dynamics across EEG frequency bands is warranted. Here, we apply data-driven machine learning (ML) models to investigate brain activity phenotypes in individuals with ID and good sleeping controls (GSCs) without imposing a priori hypotheses. Our study aims to advance understanding of sleep-related cortical activity in insomnia by exploring whether spatio-spectral sleep EEG profiles in individuals with ID of varying severity (mild to moderate/severe) (1) can distinguish them from GSCs and (2) are equally ‘trait-like’ across two nights as is the case in GSCs. We employed complementary computational approaches to address this question. First, we applied supervised ML to classify insomnia status and to identify the key features driving the model decisions. Second, we used unsupervised ML, blind to the ID status of the individual, to explore whether any spatio-spectral features separate individuals with insomnia from controls. As an additional step, we extracted the aperiodic component from the EEG spectra to determine whether the observed effects were attributable to subject-specific background activity; a critical aspect often overlooked in prior research. Aperiodic component analysis offers thus a means to isolate individual background features of brain activity across the full frequency spectrum and contributes to our understanding of interindividual variability in sleep-related brain physiology^45^.

We analyzed 20-channel EEG recordings collected over two consecutive nights at two independent research sites. The two-night protocol enhances the reliability of the findings and enables assessment of the consistency of individual sleep profiles across nights. Beyond spectral analyses, we also investigated sleep stage continuity and transitions of the sleep stages in ID and in GSCs, motivated by a few prior reports of sleep stage instability in insomnia^46,47^. Together, these analyses aim to deliver a comprehensive, multidimensional account of sleeping brain dynamics in insomnia.

## Results

**Fig. 1:**
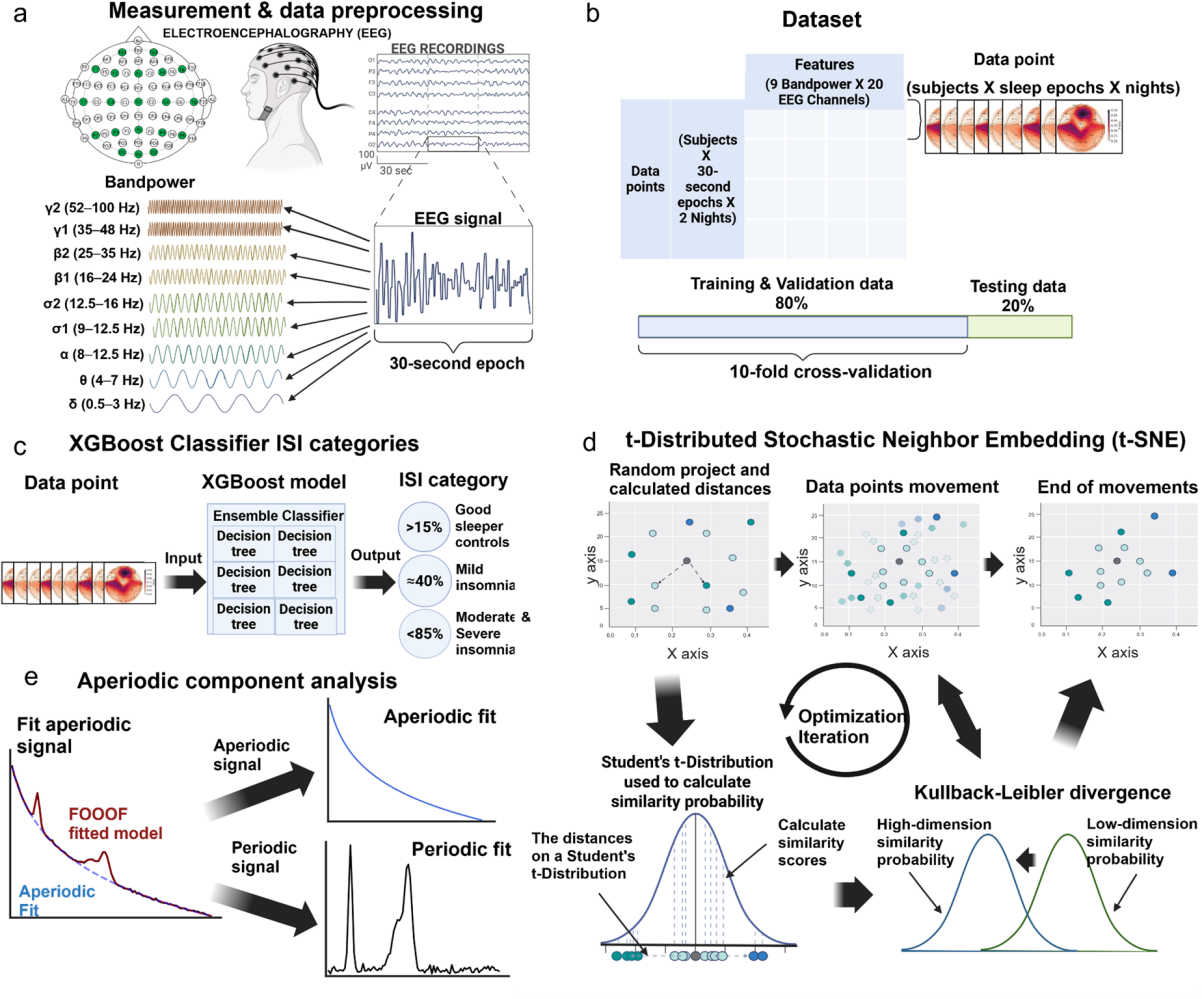
The analytical approach for the investigation of sleep EEG spectra in insomnia and in good sleeping controls. **a** We selected 20 EEG channels for the analysis and calculated power spectral density (PSD) values across nine frequency bands for each of the 30-second sleep epochs. **b** The data were split into independent samples (sleep epochs) for training (80%) and testing (20%) the supervised classification models. Features were the PSD values of the nine frequency bands and 20 channels. The resulting 180 features were used in the machine learning models. Independent samples were labeled by subject identifications and insomnia severity index (ISI) categories: good sleeper controls (GSC), mild insomnia (MI), and moderate to severe insomnia (MSI). **c** We used the XGBoost Classifier for supervised learning method to categorize sleep epochs into ISI categories based on the highest confidence output. Percentages refer to model confidence score. We employed 10-fold cross-validation to fine-tune the model’s hyperparameters to ensure optimal performance. **d** We applied t-Distributed Stochastic Neighbor Embedding (t-SNE) to visualize cortical activity during sleep epochs for dimensionality reduction. The model iteratively aligns low-dimensional and high-dimensional similarity distributions, optimizing Kullback-Leibler divergence for clear two-dimensional visualization. **e** We performed aperiodic component analysis to differentiate between aperiodic and periodic signals, allowing us to investigate the effects of insomnia on both signals. Figure created with BioRender.com.

The analytical approach is summarized in Fig. 1. The results are structured into (1) sleep EEG data description in two independent datasets (2) the analysis of sleep architecture dynamics with the probability analysis of sleep phase transitions using the Markov chain method in ID and GSCs (3) supervised classification of the EEG spatio-spectral profiles according to severity of insomnia with the XGBoost (eXtreme Gradient Boosting)^48^ classifier model (4) visualization of the EEG spatio-spectral profiles with unsupervised dimension reduction models blind to identity and insomnia severity, and analysis of similarity matrix to evaluate unsupervised model outcome, and (5) linear spectral analysis of all PSD frequencies using periodic and aperiodic component analysis according to insomnia severity.

### Description of the data

The study included 198 subjects from two independent datasets from two research sites (Dataset 1: Finland; Dataset 2: Netherlands), both comprising two consecutive overnight sleep laboratory measurements (Table 1). We used the Insomnia Severity Index (ISI)^22, 49^ to determine insomnia symptoms with cutoff scores at < 8 for good sleeper controls (GSC), 8–14 for mild insomnia (MI), and > 14 for moderate to severe insomnia (MSI). Insomnia symptoms were reported by 59% and 55% of the participants in Dataset 1 and 2, respectively. In further detail, 34% and 20% of the participants were classified having MI 25% and 34% having MSI, respectively. The difference between the datasets was not statistically significant (chi-squared test *χ*^*2*^ = 4.79, *p*-value = 0.09). In Dataset 2, we compared our cut-offs (ISI score ≥ 8; ≥ 14) against clinical diagnosis of insomnia, and found an accuracy of 91% and 85%, respectively.

### Basic sleep variables across the two datasets

Table 1 shows the basic sleep variables according to group, dataset, and night. ID-group did not have a significant main effect on total sleep time (TST) in either dataset (*p* ≥.09). Wake after sleep onset (WASO; averaged across both nights) was significantly longer in MSI (45 min) than in GSC (24 min) (*p* =.02) in Dataset 1. In Dataset 2, WASO was higher in MI (55 min, *p* =.049) and MSI (55 min *p* =.0009) relative to GSC (34 min) during Night2. In Dataset 1, Night1, N1% was higher in MI (3.3%, *p* =.0006) and MSI (3.3% *p* =.04) than in GSC (2.2%). Sleep stage bout lengths did not differ in either dataset according to night or ID-group (*p* ≥.05). Taken together, both datasets showed longer WASO across ID vs. GSC groups, but other sleep variables were not significantly different. We observed a first night effect to TST, such that it was 18 min (Dataset 1; *p* =.04) and 22 min (Dataset 2; *p* =.0006) shorter during Night1 compared to Night2. Additionally, sleep onset latency (SOL) was longer during Night1 than Night2 (27 vs. 17 min; *p* =.02). In Dataset 1, sleep stage percentages differed between the nights. During Night1, N1% (3.1% vs. 2.5%; *p* =.02) and N2% (54% vs. 52%; *p* =.04) were overall higher relative to Night2.

**Table 1:** Basic sleep variable values in two independent datasets across two consecutive nights in sleep laboratory.

		Dataset 1 *N* = 61				Dataset 2 *N* = 137			
Sleep	Nights	GSC (*n* = 25) M (± SD)	MI (*n* = 21) M (± SD)	MSI (*n* = 15) M (± SD)	Effect size ***η2***	GSC (*n* = 62) M (± SD)	MI (*n* = 28) M (± SD)	MSI (*n* = 47) M (± SD)	Effect size ***η2***
TST (min)	1	426.6 ± 37.4	407.0 ± 65.2	387.9 ± 56.3	0.08	398.6 ± 57.1	372.5 ± 69.8	376.7 ± 76.6	0.03
	2	433.4 ± 52.9	430.6 ± 55.3	415.0 ± 35.3	0.02	414.5 ± 63.7	386.0 ± 72.0	411.8 ± 58.8	0.03
	Night x ID-group				0.01				0.01
SOL (min)	1	16.6 ± 10.9	26.7 ± 29.5	27.2 ± 26.4	0.05	27.7 ± 23.9	23.2 ± 22.1	26.9 ± 24.2	0.01
	2	14.3 ± 12.8	16.7 ± 14.2	18.5 ± 13.5	0.02	28.7 ± 33.4	17.0 ± 14.7	19.0 ± 16.1	0.04
	Night x ID-group				0.01				0.01
WASO (min)	1	26.8 ± 22.6	41.0 ± 29.8	57.6 ± 47.2	0.13 *	48.9 ± 33.7	59.1 ± 44.3	56.3 ± 47.1	0.01
	2	21.0 ± 18.6	27.2 ± 26.1	32.1 ± 23.7	0.04	34.5 ± 27.9	54.5 ± 44.2	55.4 ± 36.9	0.08 **
	Night x ID-group				0.02				0.01
REM%	1	23.7 ± 5.0	24.1 ± 4.1	22.0 ± 3.5	0.04	18.0 ± 7.3	18.6 ± 7.2	17.3 ± 5.5	0.00
	2	25.8 ± 6.1	25.0 ± 4.7	23.9 ± 5.1	0.02	18.6 ± 7.3	19.3 ± 6.8	17.0 ± 7.5	0.02
	Night x ID-group				0.00				0.00
N1%	1	2.2 ± 1.2	3.8 ± 2.0	3.7 ± 2.2	0.17 **	5.4 ± 4.1	5.5 ± 4.4	5.4 ± 3.5	0.00
	2	2.0 ± 1.2	2.7 ± 1.6	3.0 ± 1.8	0.08	4.6 ± 3.4	5.4 ± 3.7	5.7 ± 3.9	0.02
	Night x ID-group				0.01				0.00
N2%	1	54.4 ± 5.8	52.6 ± 6.2	54.5 ± 10.4	0.02	55.4 ± 11.7	53.5 ± 13.0	55.6 ± 7.6	0.01
	2	52.1 ± 6.6	50.2 ± 8.2	53.9 ± 9.0	0.04	53.9 ± 10.6	51.7 ± 9.8	54.9 ± 10.2	0.01
	Night x ID-group				0.00				0.00
N3%	1	19.8 ± 5.4	19.5 ± 5.3	19.8 ± 9.2	0.00	21.3 ± 9.5	22.4 ± 10.4	21.7 ± 7.8	0.00
	2	20.1 ± 7.8	22.6 ± 6.8	19.2 ± 6.3	0.03	22.9 ± 10.2	23.6 ± 9.6	22.5 ± 8.5	0.00
	Nights x ID-group				0.01				0.00
REM bout length (min)	1	24.8 ± 4.8	24.7 ± 5.6	25.3 ± 11.5	0.00	20.1 ± 7.8	21.2 ± 8.1	19.5 ± 6.1	0.01
	2	26.0 ± 8.6	25.8 ± 6.0	27.0 ± 14.8	0.00	19.6 ± 7.5	21.6 ± 7.0	19.4 ± 6.8	0.01
	Nights x ID-group				0.00				0.00
N2 bout length (min)	1	40.6 ± 12.8	36.6 ± 10.9	38.8 ± 18.1	0.02	49.4 ± 49.0	38.6 ± 25.2	45.4 ± 43.6	0.01
	2	41.0 ± 14.5	39.0 ± 14.0	47.6 ± 19.6	0.05	40.7 ± 25.7	34.7 ± 16.3	42.1 ± 25.1	0.01
	Nights x ID-group				0.01				0.00
N3 bout length (min)	1	28.4 ± 21.3	21.8 ± 7.4	17.9 ± 7.6	0.08	25.2 ± 10.8	24.2 ± 9.1	22.5 ± 8.8	0.01
	2	22.8 ± 8.9	22.6 ± 8.7	20.1 ± 6.9	0.02	26.1 ± 11.7	26.1 ± 9.5	23.6 ± 9.3	0.01
	Nights x ID-group				0.02				0.00

The columns represent self-reported insomnia severity index (ISI) categories: good sleeper control (GSC), mild insomnia (MI), and moderate and severe insomnia (MSI). The effect size is represented by Eta squared (η2). An asterisk (*) indicates that the p-value for the One-Way ANOVA is less than 0.05, while two asterisks (**) indicate a p-value of less than 0.01. *M* Mean, *SD* Standard Deviation, *TST* Total Sleep Time, *SOL* Sleep Onset Latency, *REM* Rapid Eye Movement.

### Sleep stage transitioning probabilities according to insomnia severity

**Fig. 2:**
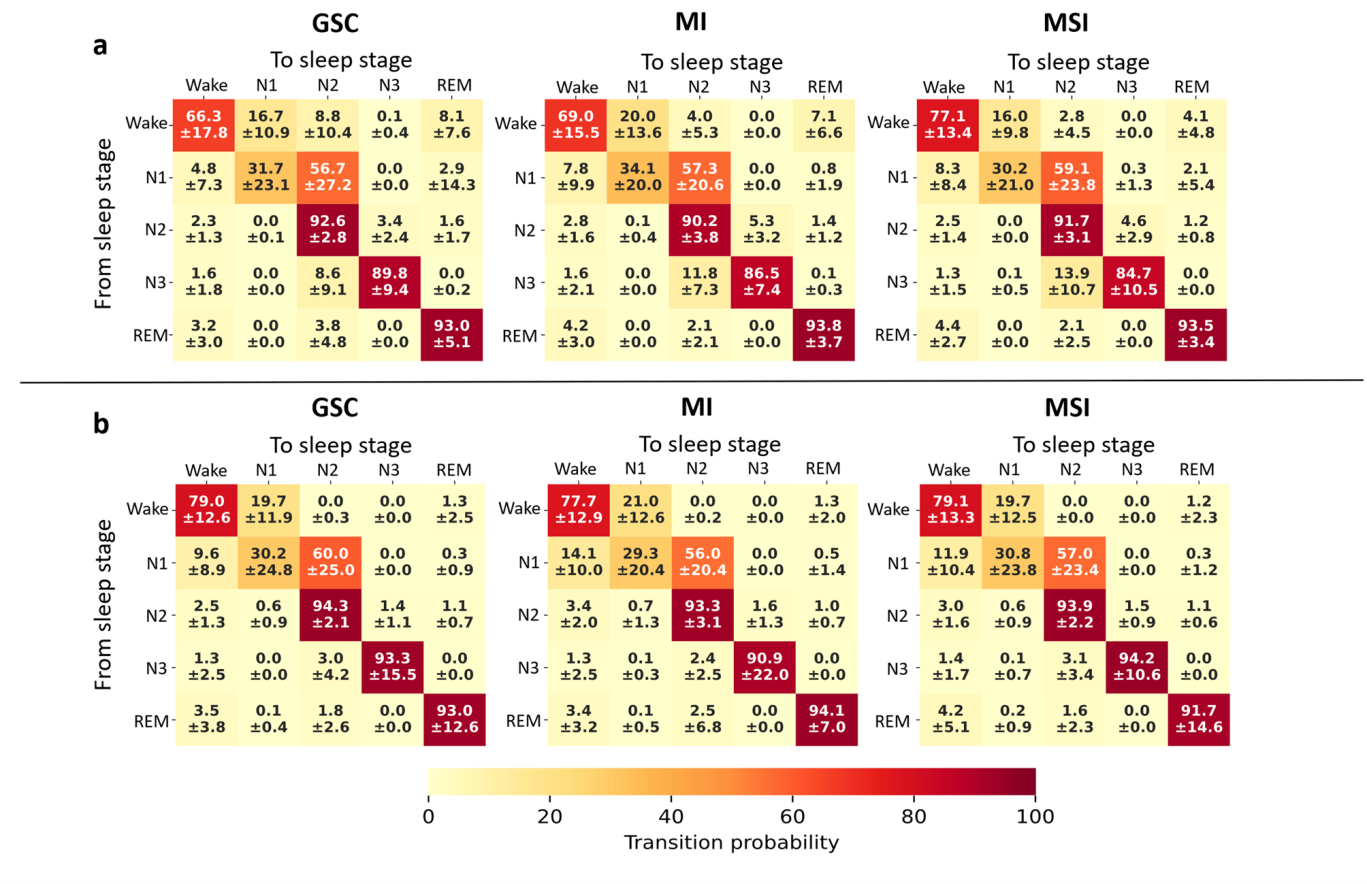
Sleep transition probability in two datasets according to the insomnia severity. The probabilities of sleep transition are presented as percentage mean and standard deviation (mean ± standard deviation) across subjects. The probability matrices are divided into insomnia severe index (ISI) category groups good sleeper control (GSC), mild insomnia (MI), and moderate and severe insomnia (MSI). Panel **a** represents Dataset 1, and Panel **b** Dataset 2. Below each matrix is a color bar: lighter yellow represents lower probability, and darker red represents higher probability. Both datasets show a lower probability of N2 stage continuity in MI than in GSC.

We utilized a Markov chain probability model to determine sleep transition probabilities according to insomnia severity (Fig. 2). To simplify the analysis of sleep transition probabilities, we present the results concatenated for each participant.

In Dataset 1 (Fig. 2a), we observed a lower probability of N2 stage continuity in individuals with MI relative to GSC (90.2% vs. 92.6%, pairwise Wilcoxon rank-sum U-test (*U)* = 627.0, *p* =.0009, *g* = –0.72). Furthermore, we found a higher Wake stage continuity probability with an increasing insomnia severity (MSI > MI: *U* = 422.0, *p* =.02 *g* = 0.55; MSI > GSC: *U* = 1044.5, *p* =.004, *g* = 0.65) and a lower N3 stage continuity probability (MI < GSC: *U* = 630.5, *p* =.001, *g* = –0.38; MSI < GSC: *U* = 416.0, *p* =.0009, *g* = –0.51). Furthermore, the transition probabilities between N2–N3 and N3–N2, indicative of a less stable N3, were higher in MI (N2–N3: *U* = 1433.5 *p* =.003, *g =* 0.68; N3–N2: *U* = 1487.0 *p* =.0006, *g =* 0.39) and MSI (N2–N3: *U* = 962.0, *p* =.04, *g =* 0.45; N3–N2: *U* = 1108.0, *p* =.0004, *g =* 0.54) relative to GSCs. The probability of transitioning from Wake stage to sleep decreased as the severity of insomnia increased (MSI < GSC: U = 395.5, *p* =.0004, *g* = –0.68; MSI < GSC: U = 488.5, *p* =.009, *g* = –0.59, respectively for Wake–N2 and Wake–REM) within Dataset 1. In Dataset 2 (Fig. 2b) the probability of transitioning from N2 to Wake increased with insomnia severity (MI > GSC: *U* = 4202.5, *p* =.02, *g* = 0.41; MSI > GSC: *U* = 7113.0, *p* =.0007, *g* = 0.41).

In sum, individuals in the ID groups in contrast to GSCs displayed a more fragmented sleep structure, as indicated by more frequent transitions from N1 to Wake, between N2 and N3, and by more prolonged wake periods.

### Classifier identifies individual insomnia EEG spectral patterns both from REM and NREM sleep epochs

**Fig. 3:**
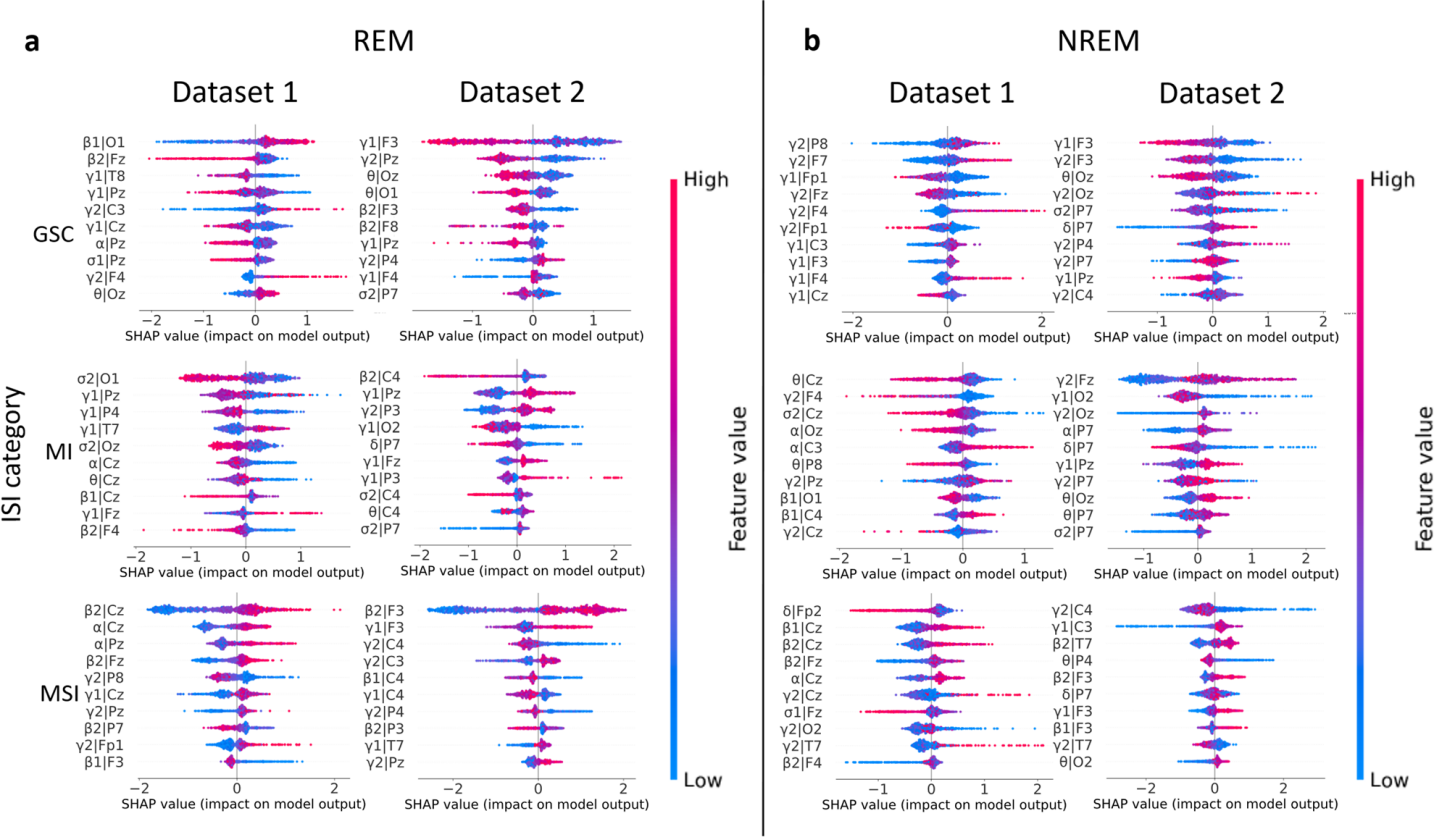
Ten most important features for different sleep stages according to the SHAP analysis performed separately for NREM and REM sleep and two datasets. SHAP (SHapley Additive exPlanations) values were derived for each classification model and ISI category, emphasizing the ten most significant features for the Intra-individual model. These values were calculated separately for each dataset as well as for the various sleep stages: **a** REM and **b** NREM sleep. In the SHAP figures, the vertical axis displays the features of the data points, while the horizontal axis illustrates the magnitude of the SHAP values. This scalar reflects the strength of the model’s classification of each data point into specific categories. Red points indicate high feature values, blue points represent low values, and purple denotes values that fall in between. The 10 most important features in the models are beta and gamma activity.

To find patterns in the brain activity that would distinguish between ID and GSC, we used the supervised learning with the XGBoost classifier to identify 30-second epochs of brain activity during sleep and to classify them into ISI categories (Table 2; Fig. 3). We ran the classifier independently in REM and NREM sleep epochs and in three distinct models. In each model, we used sleep epochs as datapoints, with features being the PSD values, and labels being the ISI category.

**Table 2:** Identification of EEG spectral patterns during NREM and REM sleep epochs with the XGBoost classifier.

		Models - Dataset 1			Models - Dataset 2		
		M1: Intra-individual	M2: Night-to-Night	M3: ISI-category	M1: Intra-individual	M2: Night-to-Night	M3: ISI-category
Model indicator		M (± SD)	M (± SD)	M (± SD)	M (± SD)	M (± SD)	M (± SD)
Accuracy (%)	NREM	98.4 (± 0.2)	66.0 (± 0.0)	35.3 (± 8.7)	98.6 (± 0.2)	57.0 (± 0.0)	36.2 (± 9.9)
	REM	97.5 (± 0.4)	65.7 (± 0.0)	34.3 (± 12.0)	97.9 (± 0.5)	59.1 (± 0.0)	33.5 (± 11.0)
Precision (%)	NREM	98.6 (± 0.2)	66.0 (± 0.0)	32.1 (± 7.9)	98.7 (± 0.2)	53.3 (± 0.0)	33.8 (± 8.6)
	REM	97.7 (± 0.4)	71.3 (± 0.0)	31.2 (± 10.3)	98.0 (± 0.5)	53.5 (± 0.0)	29.7 (± 9.9)
Recall (%)	NREM	98.0 (± 0.2)	61.8 (± 0.0)	31.6 (± 8.0)	98.4 (± 0.2)	51.8 (± 0.0)	33.2 (± 7.7)
	REM	96.9 (± 0.5)	65.1 (± 0.0)	29.9 (± 12.0)	97.4 (± 0.6)	54.4 (± 0.0)	31.2 (± 11.4)
F1 (%)	NREM	98.3 (± 0.2)	62.9 (± 0.0)	28.5 (± 7.3)	98.6 (± 0.2)	52.0 (± 0.0)	29.2 (± 8.1)
	REM	97.3 (± 0.5)	66.0 (± 0.0)	26.6 (± 9.3)	97.7 (± 0.5)	53.0 (± 0.0)	26.1 (± 8.9)

The table presents the performance results of supervised classifiers utilizing three model versions tested on both rapid eye movement (REM) and non-REM (NREM) data across two datasets. Key performance metrics -accuracy, precision, recall, and F1 score - were computed from the test data, with definitions provided in the supplementary materials. Model Intra-individual achieved an accuracy of 97–98%. Model Night-to-Night had an accuracy of 57–66%. Model ISI-category reached an accuracy of 33–36%. *ISI* insomnia severity index, *M* mean, *SD* standard deviation, *F1* harmonic mean of the precision and recall, see Supplement S2.

We applied supervised classification in three different models that diverge in the use of samples and participants in the allocation to training and testing datasets. The purpose of the three different models is to examine how changes in the training data impact the model’s performance and provide additional information for the model’s decision-making. In the first supervised classification model (Model 1, *Intra-individual Model*), we used pooled data from two nights and randomly divided datapoints into two independent samples, 80% allocated to training and 20% to testing. Model 1 classification accuracy of insomnia severity was 98–99%.

In the second model (*Model 2; Night-to-Night Model*), we kept the nights separated and trained the classification model with the sleep data from Night1 and tested it using Night2 data. Model 2 accuracy was 66% for both NREM and REM in Dataset 1 and 55% and 57% in Dataset 2, respectively, indicating higher nightly fluctuation in the individual brain activity patterns in Dataset 2, but also a level of stability in intraindividual brain activity.

In Model 3 (*ISI-category Model*), we tested the across-participant generalization of the decoder by dividing the participants randomly into two groups, 80% allocated for the training, and 20% for testing. This model aimed at identifying insomnia severity without having participants’ own data in the training. The accuracy of Model 3 ranged between 30 and 40%, corresponding to a random classification.

In summary, all the model outcomes were converging across the two datasets and across REM and NREM sleep. While we found high accuracy in classifying ID, this was only seen in Model 1, where the training and testing were performed with the same individuals for one night. Using another night to test (Model 2) lowered the accuracy. Finally, if the testing was done without having the same individuals’ samples in the training phase, the accuracy dropped to chance level (Model 3).

### Feature importance in classification decisions

A SHapley Additive exPlanations (SHAP)^50^ analysis was performed on the outcomes of Model 1 (*Intra-individual Model*) to identify the feature importance in the models’ classification decisions (Fig. 3a for REM sleep, Fig. 3b for NREM sleep). Since the feature importance of different EEG channels varies over time, reflecting the complexity of the data, the analysis was repeated 100 times for each supervised classifier. This ensured the consistency of the feature importance results.

The SHAP results indicate that the most prevailing features influencing the models’ decisions are characterized by high-frequency (beta and gamma bands) activity. In REM sleep (Fig. 3a), the beta band accounts for 23% and the gamma band for 50% of the top 10 feature importance. In NREM sleep (Fig. 3b), the respective shares are 15% for the beta band and 53% for the gamma band. If we take only the first and second decision features, these bands dominated entirely in Dataset 2 and were highly prevalent also in Dataset 1. We also observe that the alpha and sigma bands accounted for 17% of REM sleep and 13% in NREM sleep, making them the most prevalent decision features after the beta and gamma bands. The model decision features were alike across the groups (GSC, MI, MSI).

In sum, EEG frequencies indicating high cortical arousal (beta and gamma bands) were the important features in the EEG classification decisions, irrespective of the ID status.

#### Unsupervised analyses reveal trait-like patterns of brain activity that are not associated with insomnia severity

**Fig. 4:**
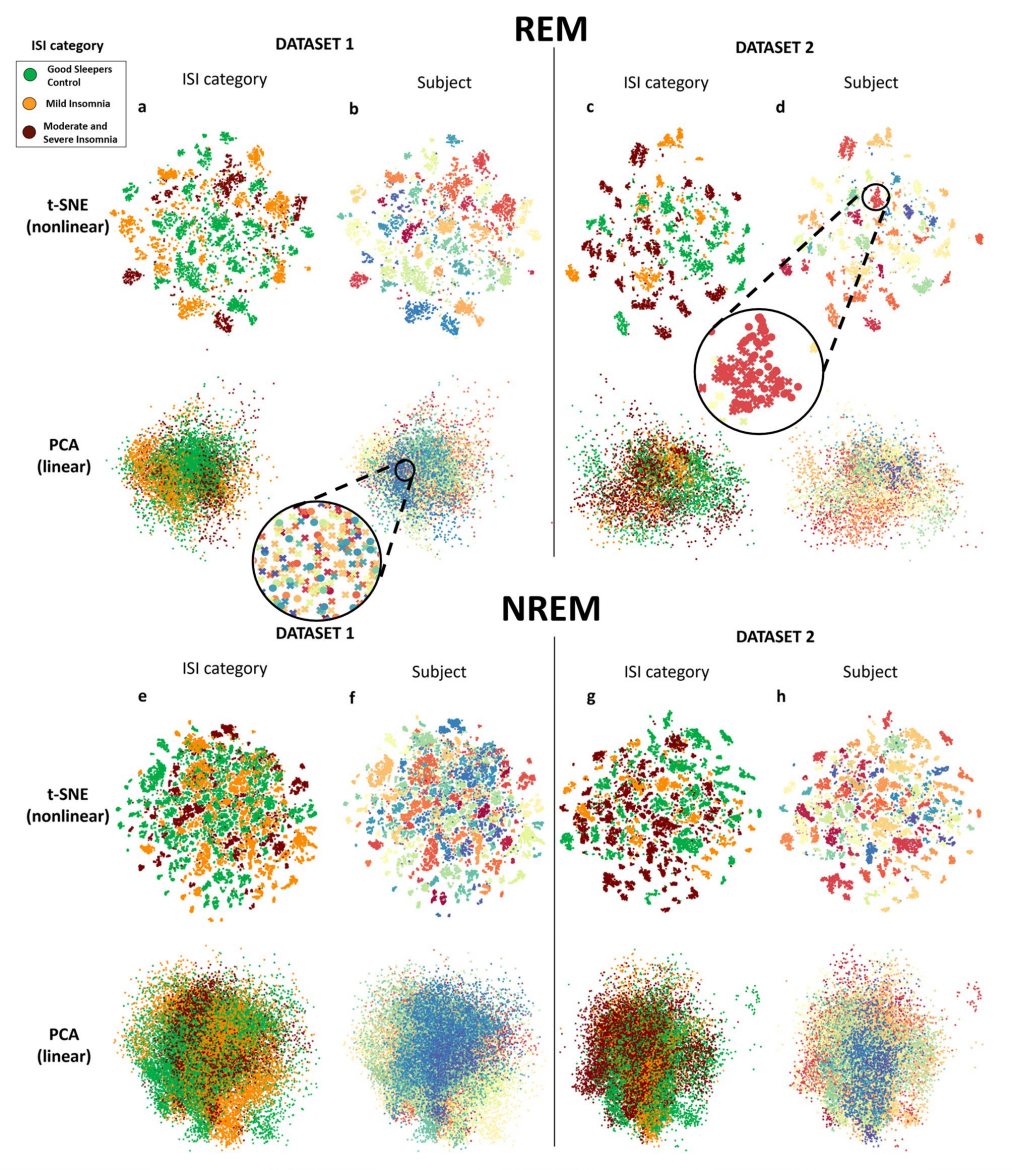
Unsupervised methods identify unique EEG spectral patterns during REM and NREM sleep. The t-SNE visualization illustrates the PSD values for brain waves during REM (**a**–**d**) and NREM (**e**–**h**) sleep as a scatterplot. In this visualization, data points are color-coded according to the insomnia severity index (ISI) category (**a**, **c**, **e**, & **g**) and by subject identification (Subject) (**b**, **d**, **f**, & **h**). A similar analysis was conducted using principal component analysis (PCA), a linear dimension reduction method, in contrast to t-SNE, a nonlinear method. The clusters of data points represent similarities among the samples. The data points are depicted as either circles or crosses, indicating their correspondence to Night1 and Night2 data, respectively. Distinct colors uniquely represent each subject’s identity and insomnia severity group. The results show that t-SNE reveals trait-like patterns in individual fingerprints of subjects based on brain wave activity during REM and NREM sleep.

To elucidate state and trait-like patterns of brain wave activity in REM and NREM sleep, we applied the t-distributed stochastic neighbor embedding (t-SNE) method and principal component analysis (PCA) to the absolute values of EEG power spectra computed in 30-second epochs across Night1 and Night2. The analyses were fully blind to subject identity, night order, and insomnia severity, and were performed independently in the two datasets. The resulting independent samples of sleep epochs were color-labeled according to the ISI categories and the subjects’ identifications, as shown in Fig. 4.

Figure 4 shows that the sleep epoch independent samples form clusters according to individual subjects (Fig. 4b, d, f and h) in both datasets, such that Night1 and Night2 from the same identity cluster are mostly appearing together. Figure 4a, c, e and g show that there is no observable clustering according to insomnia severity. Thus, the visual inspection indicates that sleep-related cortical activity is dominated by individual variability across the subjects, aligning with the statistical outcomes from the XGBoost classification model.

A qualitative comparison of the visualizations of the t-SNE and PCA models in REM and NREM sleep confirmed that the non-linear t-SNE generates clear identity-specific clusters of EEG spectral samples, while the linear PCA does not (Fig. 4). Standardizing the EEG data did not affect the PCA results, and there was no excessive muscular activity that could have influenced the findings (Supplement S4).

**Fig. 5:**
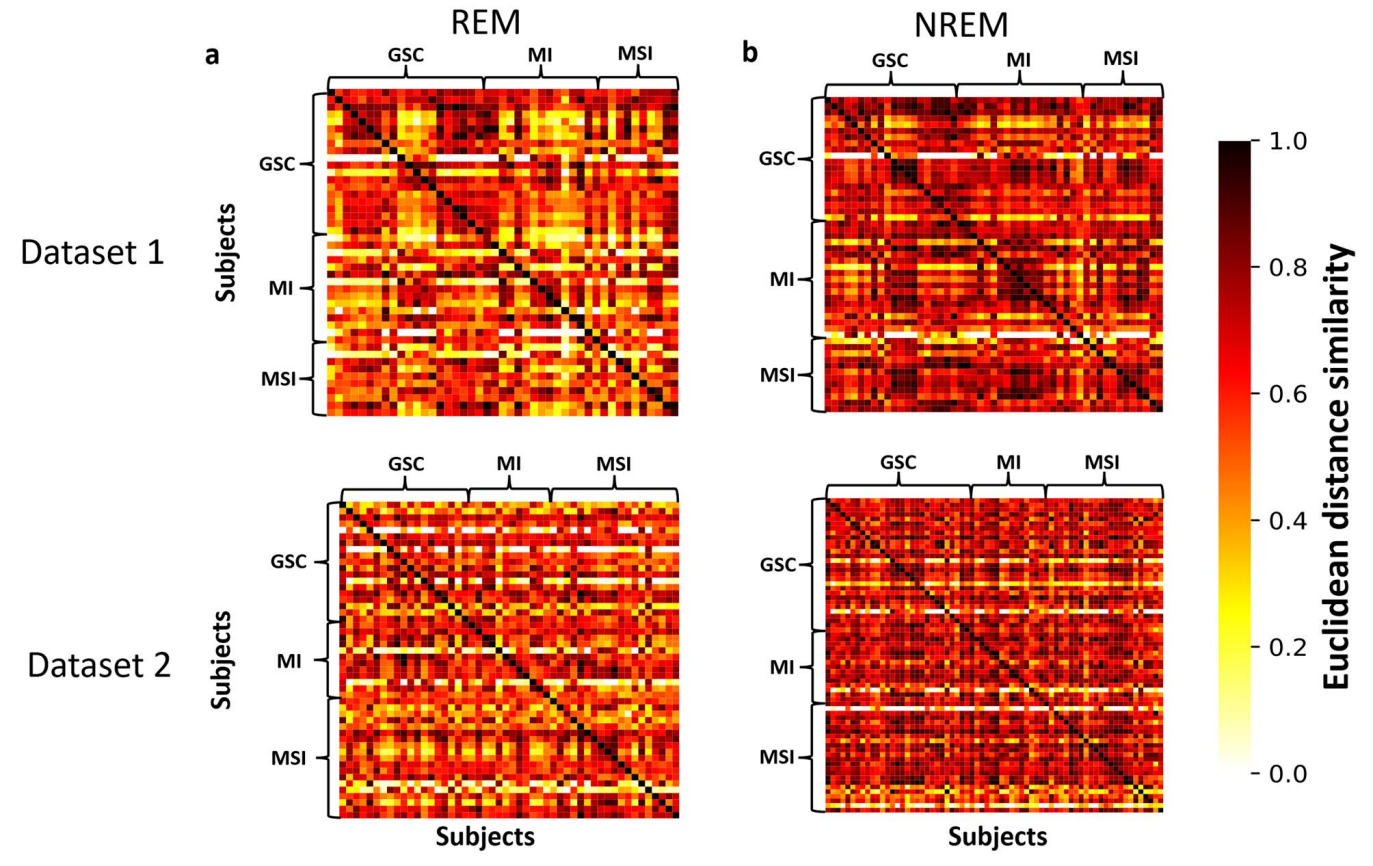
The similarity matrix revealed subject-specific brain wave activity patterns during REM and NREM sleep. The similarity matrix is based on a separate analysis of Dataset 1 (top panel) and Dataset 2 (bottom panel). The matrix illustrates the Euclidean distance similarity between the power spectral density (PSD) values of **a** REM sleep and **b** NREM sleep, one pixel corresponding to a 30-second epoch. The subjects are arranged in the same order from top to bottom and left to right. Each pixel in the similarity matrix represents the similarity of PSD values between and within (the diagonal line) subjects. A lighter color indicates lower similarity, while a darker color represents a high similarity in the PSD values. Additionally, subjects are categorized according to their insomnia severity index (ISI), with groups labeled as good sleeper controls (GSC), mild insomnia (MI), and moderate to severe insomnia (MSI).

Next, to verify the statistical validity of the observed t-SNE clusters on subject identity, we computed a similarity matrix of subjects’ EEG signal activity between two randomized sets of sleep EEG data from each subject (Fig. 5). A higher similarity of EEG spectrum within the subject is shown in diagonal lines (darker red) both in REM and NREM data. We found no consistent within-group similarities or between-group differences across ISI-defined groups in either dataset or across sleep stages.

To complete the analysis, we also conducted linear comparisons of the group average between ID and GSC in the EEG power across distinct EEG frequency bands. These analyses showed no significant differences between the groups either in REM or NREM sleep when averaged across channels (Supplement S2). Furthermore, there were no interactions related to insomnia in either NREM or REM over the two nights (all *p* ≥.11). We also analyzed both relative and absolute PSD, adjusting for sex and age (Supplement S2). There were no differences in sleep stages or nights between ID and GSC groups (all *p* ≥.07).

### Periodic and aperiodic signal analysis according to insomnia severity

**Fig. 6:**
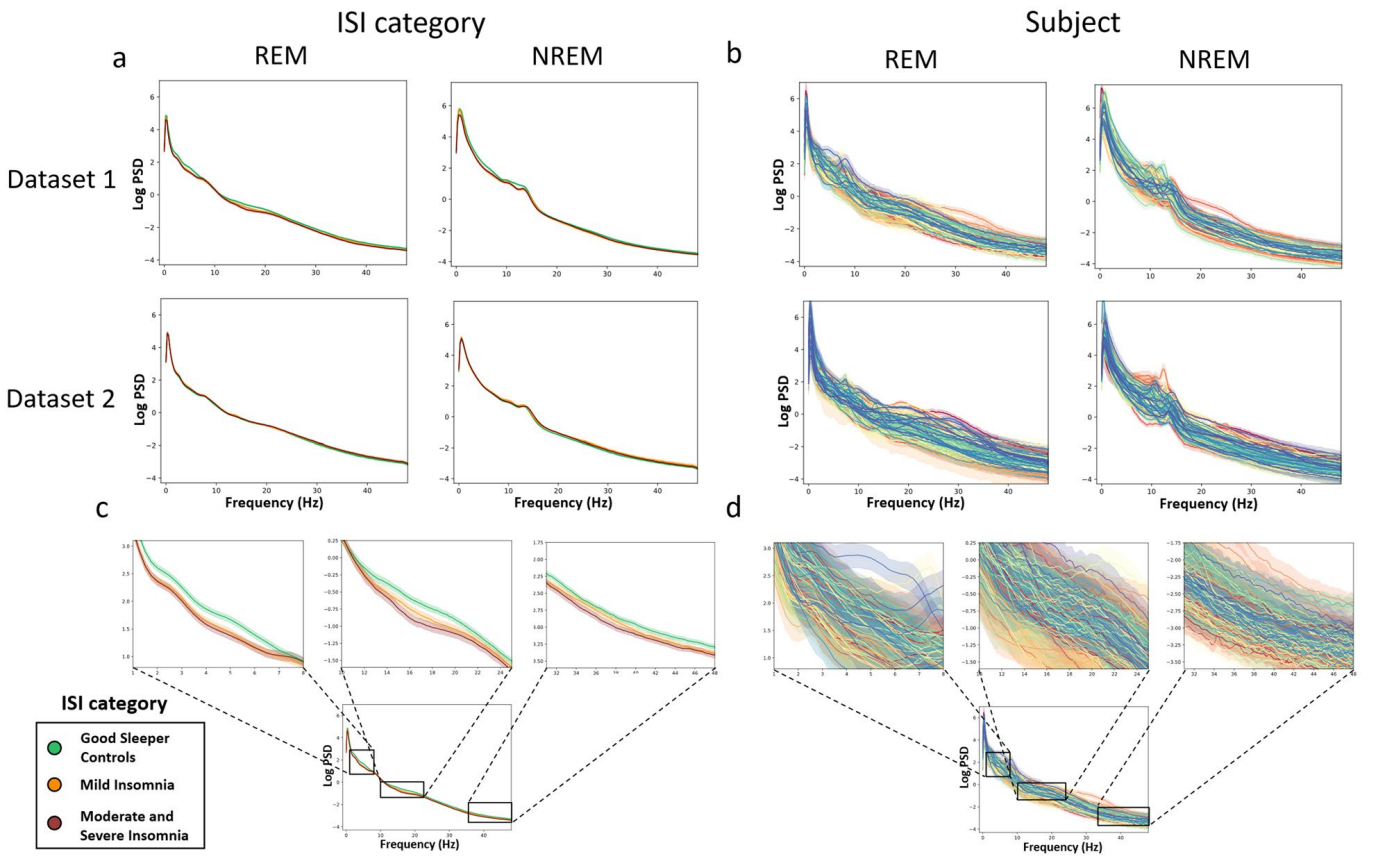
The periodogram reveals that subject-specific EEG spectra during REM and NREM sleep is larger than insomnia in any specific frequency bands. Periodograms during REM and NREM sleep across insomnia severity (**a** & **c**) and subject identification (Subject) (**b** & **d**). Dashed boxes indicate zoomed-in areas, while dashed lines mark specific sections of the periodogram. Distinct colors uniquely represent each subject’s identity and insomnia severity group. Panels c and d are from REM sleep in Dataset 1. The dark lines indicate the mean values, and the light area around dark line represents the 95% confidence interval. The confidence interval is calculated using the seaborn visualization package via a nonparametric bootstrap approach.

We created brain activity periodograms averaged across channels using the ISI category and subject identity (Fig. 6). The periodograms show that insomnia severity (Fig. 6a and c) is not reflected in the brain activity patterns in either NREM or REM sleep. In Dataset 1, the total log PSD values during REM sleep of the insomnia group (Fig. 6c upper panel) are lower than GSC groups, but not significantly (Tukey HSD post-hoc test MI: *T* = − 0.93, *p* =.62, hedges’ *g* = − 0.26; MSI: *T* = − 2.33, *p* =.06, *g* = − 0.76). However, this pattern does not occur in Dataset 2, where the total log PSD values are nearly identical across insomnia severity (REM: Bayes Factor *BF*_*01*_ > 1.0; NREM: *BF*_*01*_ > 3.4). Yet, the individual variability was again large (Fig. 6d), and more than seven times greater variance than the observed differences between the ISI groups (REM: a One-way ANOVA *p* =.98, *F* (2, 112) = 0.018; NREM: *p* =.88, *F* (2, 112) = 0.130) in Dataset 2. The periodograms showed similar PSD patterns across the two independent datasets.

**Fig. 7:**
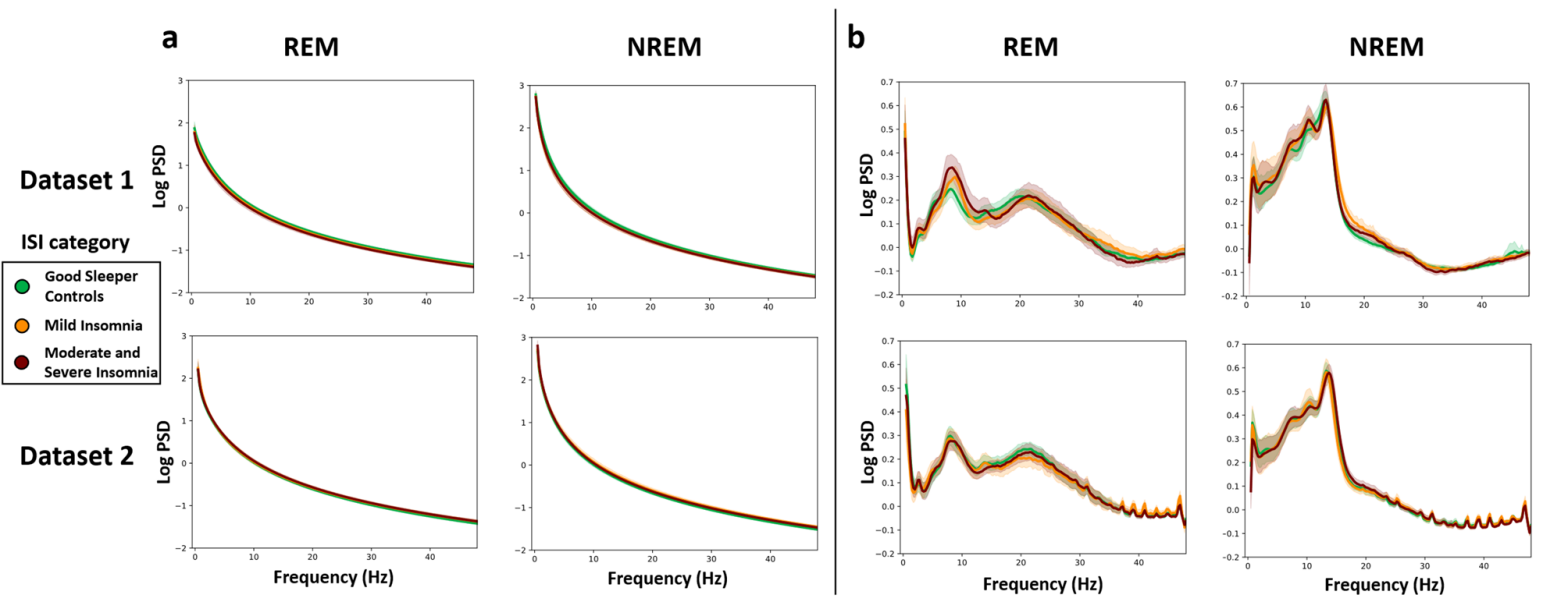
The aperiodic component analysis showed that both aperiodic and periodic signals exhibited similar behavior across the datasets in REM and NREM. The periodograms (**a**) display REM and NREM sleep data with the aperiodic component removed through periodic component analysis. The scatterplot (**b**) shows aperiodic parameters, with the exponent represented on the vertical axis and the offset on the horizontal axis. Insomnia severity is categorized by the insomnia severity index (ISI): blue for good sleeper controls (GSC), orange for mild insomnia (MI), and green for moderate and severe insomnia (MSI). The Figures at the top use Dataset 1, while those at the bottom use Dataset 2. The dark lines indicate the mean values, and the light area around dark line represents the 95% confidence interval. The confidence interval is calculated using a nonparametric bootstrap approach.

We performed an aperiodic component analysis averaged across EEG channels to investigate whether insomnia-specific brain activity emerged after eliminating the aperiodic components of the EEG data, which may include subject-specific elements. We removed wire artifacts from the EEG spectrum through interpolation and adjusted the FOOOF model fitting to the 0.5–100 Hz range. For the analysis, we focused on the 0.5–45 Hz range to highlight key results. We obtained periodic and aperiodic parameters, created an aperiodic fitted spectrum using aperiodic parameters, and subtracted this from the original spectrum to obtain the periodic spectrum. Our study focused on the parameters of aperiodic components, including the offset/intercept and exponent/slope, as well as the periodograms derived from the EEG signal, with the aperiodic component removed. The results of aperiodic component analysis are shown in Fig. 7.

Figure 7a displays the spectra of the aperiodic component. They did not vary substantially according to insomnia severity in either dataset. The median intercept values were 2.0 (standard deviation SD = 0.4) and 2.1 (SD = 0.5) during REM, while during NREM sleep, the median values were 2.4 (SD = 0.4) for both Dataset 1 and Dataset 2. The median intercept during NREM was approximately 0.4 higher than during REM sleep in both datasets. The median slope parameter values were both 2.3 (Dataset 1: SD = 0.2 and Dataset2: SD = 0.3) during NREM, and median values were 2.0 (SD = 0.2) and 2.1 (SD = 0.3) during REM sleep, Dataset 1 and Dataset 2, respectively. The median slope during NREM was also approximately 0.3 higher than during REM sleep in both datasets.

Figure 7b illustrates the periodogram after removing the aperiodic component from both REM and NREM sleep data. For REM and NREM sleep, both datasets exhibit similar patterns and do not reveal any statistically significant insomnia-specific activity patterns (a One-way ANOVA test all *p* >.05).

## Discussion

Our findings demonstrate that brain activity during sleep is characterized by robust, individual-specific EEG signatures, while no insomnia-specific EEG power spectrum alterations were detected. The signatures proved to be trait-like across two nights, similarly in individuals with insomnia and good sleepers. Although previous studies have reported individual patterns in sleep EEG^33–35,51^, our results provide novel evidence that these signatures are specifically anchored in high frequency cortical activity. In contrast to prior studies^1,16–21,28^, we analyzed EEG power without the models knowing participants’ identities, allowing the clusters of sleep epochs to be based solely on unique brain activity features over two nights. The individual EEG signatures proved robust: significant interindividual variability in the EEG power spectrum persisted even after the assessment of periodicity, offering an alternative means of isolating subject-specific background features in the EEG spectra^45^. Finally, model replication across two independent datasets from separate research sites showed high convergence, underscoring the generalizability of these findings.

In parallel with the ML approach, we conducted traditional linear analyses and found no significant group differences in EEG spectral metrics across two nights between individuals with ID and GSC - a topic that remains contentious in the literature^1,16–21,28^. These findings may also suggest that individual variability in EEG power signatures may obscure group-level features linked to insomnia. We demonstrated that a trained model achieved a remarkably high accuracy (98–99%) in classifying insomnia from EEG spectra, but this performance was only attainable on using data from the same individuals and same night. Accuracy declined substantially (to 57–66%) when testing across nights within individuals and fell to chance levels (26–36%) when training and testing involved different individuals. We speculate that the high classification performance reported in some previous studies was driven mainly by the same participants being present in both training and testing data, and it is unclear how generalizable these models are.

Both independent datasets revealed consistent patterns of marked interindividual variability in EEG activity, as confirmed by ML analyses. This variability was not captured in standard linear methods such as PCA, suggesting that non-linear techniques are better suited to disentangle the multidimensional interdependencies of EEG spectral features. These properties may contribute to inconsistent findings regarding insomnia-related cortical hyperarousal in prior studies^3,12,27^. Equally, this interindividual variability may explain why efforts aimed to discover insomnia-related functional connectivity features have resulted in very heterogenous findings^52^. Finally, while EEG power spectra offer valuable insight into sleep neurophysiology, there are other EEG features that are of interest for insomnia. For example, the continuity of sleep stages is often affected in ID. Consistent with previous research^28^, we observed insomnia-related alterations in sleep stage transitioning, including increased transitions from N1 to Wake, longer wake periods, and reduced stability of NREM sleep in N2 and/or N3^47^. In contrast, REM sleep continuity was preserved in individuals with insomnia, showing no signs of increased fragmentation compared to good sleeper controls^46^. 

We found that high-frequency brain activity during sleep is a key feature contributing to individual signatures of sleep. However, the functional significance of beta- and gamma range activity remains incompletely understood. Numerous studies have implicated gamma activity in neural synchronization processes. For example, during wakefulness, high frequency brain activity has been associated with the synchronization of distributed brain areas involved in visual processing^53^ and with the coordination of neural dynamics at the brain circuit level^54^. During sleep, numerous studies have associated gamma-band activation with brain synchronization and memory consolidation processes^55–57^.

### Limitations

Overall, the operationalization of insomnia is not consolidated, as even the clinical criteria vary across the most used diagnostic classification systems, DSM-IV^58^ and V^59^, ICSD-R^60^ and 3^61^, and ICD-10^62^ and 11^63^. In practice, insomnia detection and diagnosis methods rely mostly on subjectively reported indicators such as threshold classifications from the ISI, also used in the current study. However, we performed a cross-validation between the ISI score and clinical diagnosis of insomnia in our data and found a high agreement rate.

Sleep EEG activity above 50 Hz has been suggested to associate with muscle activity and the residual 1/f drop-off, rather than being solely brain-driven. We investigated the muscle activity from EMG signals and diminished the probability of muscle activity as being the origin of high frequency activity in our datasets.

It is also worth noting that the activity in the different frequency bands during sleep may not be equally expressed at all brain locations. For example, a previous study has shown that rhythms in the gamma range (> 30 Hz) were significantly reduced both in N2 and N3 compared to wake, but this reduction was most predominant in the prefrontal cortex^64^. This study did not account for the source of localization, which may play a role also in the individual signatures of sleep oscillations. We also acknowledge that the variation in insomnia classification in the night-to-night model may have been influenced by the first night effect of sleep laboratory measurements. Nevertheless, it shows that sleep oscillations related to insomnia are prone to within-subject variation over time.

## Conclusions

We demonstrate here the existence of individual-specific EEG signatures with trait-like stability over two nights. We discovered that high-frequency brain activity during sleep was central in distinguishing these individual sleep EEG signatures in the non-linear analyses. Although this activity can be indicative of cortical hyperarousal^1, 16–21^, it was not specific to insomnia in either dataset, but rather best captured individual variability. While insomnia remains a significant public health concern, our findings suggest that tracking EEG continuity and disruptions at the individual level - rather than relying on group comparisons – may offer better insight into the disorder.

Addressing the complexity of sleep-related brain activity may require repeated EEG measurements within individuals to detect temporal changes over time. This approach enables the identification of unique intra-individual dynamics, mitigating the confounding effects of interindividual variability. Our suggested approach seems necessary to understand underlying mechanisms of insomnia and ultimately support the development of personalized treatments.

## Methods

All statistical analyses were conducted using Python (version 3.8.19). All plots were generated with the Matplotlib package (version 3.7.5)^65^ and Seaborn package (version 0.11.2)^66^.

### Participants

#### Dataset 1

61 voluntary participants (82.0% female; M age = 31.5, SD = 7.0 years) enrolled for a study on insomnia in Helsinki, Finland. Half of them were invited with self-experienced insomnia fulfilling the diagnostic criteria ICD-10^63^, and half identified themselves as good sleepers. The exclusion criteria included sleep apnea, narcolepsy, or the use of medication that significantly affects sleep due to sedative properties or impact on sleep architecture. The ethics committees at the University of Helsinki approved the study protocol. Written consent was collected from each participant at the beginning of the study in accordance with the Declaration of Helsinki. The recruitment procedure was validated upon completing the ISI questionnaire^22,49^, which measures insomnia symptoms experienced over the past two weeks and was used as the primary assessment method of insomnia severity. The participants underwent sleep laboratory measurements over two consecutive nights to measure polysomnography (PSG). On the day of the experiment, participants were required to wake up by 8 am and stay awake until the experiment started. They were prohibited from drinking coffee after 1 pm and avoiding any intoxicants throughout the day.

#### Dataset 2

137 voluntary participants (72.3% female; M age = 47.6, SD = 14.4 years) enrolled in various studies at the Sleep and Cognition lab of the Netherlands Institute for Neuroscience for two consecutive PSG nights. Studies had comparable inclusion/exclusion criteria and overnight protocols but varied in daytime experimental procedures. While participants either received an insomnia disorder diagnosis according to current DSM and ICSD criteria or qualified as good sleeper controls, we used available ISI scores for consistency across datasets. Individual study protocols were approved by the ethics committees of either VU Medical Center or University of Amsterdam. All participants adhered to their own habitual sleep timing, gave written informed consent in accordance with the Declaration of Helsinki, and were paid for participation. Dataset 2 was utilized to compare the consistency of the outcomes obtained from Dataset 1.

### Data collection and preprocessing

#### Dataset 1

High density EEG was recorded using 128-channel EEG set and software by Brain Products (Brain Vision LLC, https://brainvision.com), connected to an actiCHamp Plus amplifier (sample frequency 1000 Hz). The ground electrode was placed at Fpz. Electro-oculograms (EOGs) and electromyograms (EMGs) were measured with electrodes from one of the EEG bundles: EMG was attached to the chin, EOG1 to the right cheek, and EOG2 kept in the cap above the left eye. The online reference electrode was FCz.

#### Dataset 2

High density EEG was recorded using a 256 Ag/AgCl channel LTM HydroCel EEG net (Electrical Geodesic Inc, Eugene, OR) connected to a Net Amps 300 amplifier (input impedance: 200 MΩ, A/D converter: 24 bits, sample frequency 1000 Hz). The ground electrode was placed between CPz and Pz, with Cz serving as the online reference. Electrode impedances were kept below 100 kΩ. Additional bipolar physiological signals collected chin and leg EMG, ECG, and respiration. Both datasets were converted to a PSG montage of standard EEG, EOG and EMG for visual scoring of sleep stages and arousals. Sleep data were classified by experienced sleep scorers in 30-second epochs as wake, N1, N2, N3, or REM sleep stages, according to the guidelines of the American Academy of Sleep Medicine (AASM) Manual^67^. All signals were band-pass filtered by AASM-compliant filters.

From both datasets, we selected the same 20 EEG channels (Fp1, Fp2, F3, F4, C3, C4, P3, P4, O1, O2, F7, F8, T7, T8, P7, P8, Fz, Pz, Oz and Cz) for the analyses, ensuring even distribution across the scalp area. and incorporating both datasets. The signals were digitally filtered with offline band pass filter of 0.5–100 Hz. A Hamming windowed sinc zero-phase FIR filter was used as a filter. The cutoff (–6dB) was 0.25 Hz and 112.5 Hz, respectively. A notch filter (50 Hz) was used to remove signal interference from the power source. Signal filtering was done by using MNE-Python (version 1.6.1)^68^.

The PSD values were calculated using the “welch” function in SciPy (version 1.10.1)^69^, where the window length was 4000 samples (4 s), and the bins overlap was 50% with 0.25 Hz resolution. The values obtained from the bins were averaged over one sleep epoch. The PSD values were changed into final PSD values by taking the natural logarithm. Nine different frequency bands were used: delta (0.5–3 Hz), theta (4–7 Hz), alpha (8–12.5 Hz), low sigma (9–12.5 Hz), high sigma (12.5–16 Hz), low beta (16–24 Hz), high beta (25–35 Hz), low gamma (35–48 Hz) and high gamma (52–100 Hz). We wanted to use both the alpha and low sigma frequency bands to assess the differences in the effects of slow spindle activity compared to only alpha activity. PSDs for these frequency bands were obtained from NREM (N2 + N3) and REM sleep.

For both Dataset 1 and 2, we categorized the ISI based on established cutoffs^22,49^. We categorized the ISI scores into three categories: a score of < 8 indicates GSC, 8 to 14 indicates MI, 15–21 denotes moderate insomnia, and a score > 21 reflects severe insomnia. To improve clarity, we combined moderate and severe insomnia into MSI category.

### Channel artifacts and outlier detection

We removed epochs containing artefactual data with a method consisting of steps:

Signals were passband filtered from 0.5 to 145 Hz, resampled to 200 Hz; and divided into 30-second epochs. The amplitude differences between adjacent signal samples were calculated, and the epoch-wise amplitude differences were averaged. Finally, any amplitude difference greater than 5 microvolts between average adjacent samples within a sleep epoch was classified as artefactual and excluded from spectral analyses. Artifact epochs were excluded from 69% of Dataset 1 and 88% of Dataset 2. Additional details can be found in Supplement S1.

### Sleep statistics

The YASA toolbox (version 0.6.4)^70^ was utilized to calculate sleep variables from the hypnogram. We employed Pingouin (version 0.5.5)^71^ to conduct a one-way ANOVA to compare the traditional sleep EEG variables between the ISI groups and across the two nights. If the p-value threshold is below 0.05, we applied the Wilcoxon rank-sum statistical test to maintain non-parametric assumptions about the data distribution. Additionally, we utilized Hedges’ g effect size to quantify the difference effect. We used the Markov-chain method^72^ with a chain length 960 to calculate the sleep stage transition probabilities, and Wilcoxon rank-sum test and Hedges’ g effect size to compare the transition probabilities across the insomnia severity groups. The probabilities are presented in a heatmap, with a color scale in percentages from zero to hundred, from light yellow to dark red.

### Supervised classification model

We utilized the XGBoost (eXtreme Gradient Boosting)^48^ classification model from the gradient boost family algorithm for supervised classification. We selected this model due to its versatility and its ability to manage accurate data with missing values without compromising performance. Log-transformed absolute PSD values from nine frequency bands of 20 different EEG channels were used as features for the XGBoost model. For our data allocation, 20% was designated for testing while 80% was reserved for training. We performed a 10-fold cross-validation on the training data to determine the best parameters for the XGBoost model (version 2.0.3). The model was trained and evaluated 100 times. We opted for the “auto” method for tree construction algorithm in XGBoost. The learning rate was set to 0.3, and gamma was set to 0. The maximum depth of the trees was limited to 10, and the L1 regularization term on weights was also 0.

We calculated the confusion matrix (in Supplement S3) and feature importance, which was determined using the SHapley Additive exPlanations (SHAP) (version 0.44.1) analysis to identify reasoning of the classification decisions. SHAP is based on a game theory context that can explain the effects of features used by ML models on the model’s classification. This method accounts for both the positive and negative contributions of features to the classification outcomes. To reduce computational complexity, we used 1000 randomly selected data points from the test data to visualize the results of the SHAP analysis. We only included Model 1 in the results because the other models performed poorly and produced random outcomes, which could have biased the findings. Therefore, those models were excluded from the SHAP analysis.

### Dimension reduction methods

The t-distributed stochastic neighbor embedding (t-SNE)^73^ method was utilized as a nonlinear dimension reduction technique to examine the multidimensional dynamics of brain waves. To compare with t-SNE and to enhance computational efficiency, we used principal component analysis (PCA)^74^ as a linear dimension reduction method for initialization. It is important to note that the use of PCA for initialization does not alter the results obtained from t-SNE. We input the power spectral density (PSD) values featuring nine frequency bands from 20 different EEG channels into dimension reduction models. Both t-SNE and PCA were employed to reduce the 180-dimensional feature set to two dimensions, which were visually represented using a scatter plot. The implementation of both methods relied on scikit-learn (version 1.3.2)^75^. In our t-SNE implementation, the distance metric used was Euclidean distance, with a learning rate set to auto’ and a maximum of 1000 iterations. All t-SNE figures were created with a perplexity setting of 30.

### Similarity matrix

Half of the sleep epoch pairs were randomly selected based on PSD values from nine frequency bands across 20 EEG channels, categorized by sleep stage and participants. The Euclidean distances were calculated to determine subjects’ intra- and inter-variability. Euclidean distances were normalized with Min-Max normalization and change their values to negative, which then increased by one to convert Euclidean distance to similarity (the smallest Euclidean distance approaches one and the longest distance approaches zero). This method’s calculation is described by Eq. (1):

1 $$\:{S}_{e}\left({X}_{i},{X}_{j}\right)=1-\mathrm{n}\mathrm{o}\mathrm{r}\mathrm{m}\left(\sqrt{{X}_{i}{X}_{i}^{T}-2\left({X}_{i}{X}_{j}^{T}\right)+{X}_{j}{X}_{j}^{T}}\right),$$

where a dataset $\:{X}_{N}$ consists of *N* elements ($\:i\ne\:j$) the “norm” refers to the Min-Max normalization function and $\:{S}_{e}$ represents the Euclidean distance variable used to define similarity within a similarity matrix. Normalization aims to convert Euclidean distances into similarity values, which can be represented as numbers between 0 and 1. This approach simplifies the interpretation of results compared to using high distance values.

### Periodic and aperiodic component analysis

We estimated periodograms between 0 and 48 Hz (0.25-Hz resolution) for each subject’s sleep epochs. Samples were averaged per night, for REM and NREM sleep separately.

Aperiodic component analysis was performed using the fitting oscillations & one over f (FOOOF) toolbox version 1.1.0^45^, which separates the periodic and aperiodic signals. Before extracting the aperiodic component from the signal, line noise is removed by interpolating around the peaks of the line noise. This preprocessing helps reduce the influence of the fractal (1/f) noise and enhances the subsequent fitting process of the FOOOF.

The FOOOF fits the aperiodic signal using a formula that defines the slope and intercept parameters for the aperiodic component in relation to the original signal. The aperiodic signal created with this formula is then used to extract the remaining periodic signal. The algorithm identifies the central frequency, peak power, and peak bandwidth of the periodic signal peaks. Peaks are defined based on a noise threshold derived from the aperiodic signal; any values above this threshold are classified as peaks. A Gaussian is fitted according to these peak parameters, and this fitted Gaussian is then subtracted from the periodic signal.

Following this, the noise threshold is recalibrated, and peak parameters are re-evaluated with another Gaussian fit. The standard deviation of the residuals is recorded at this stage. The process continues until no more peaks are detected in the periodic signal above the noise threshold. All collected peak parameters are then used to fit a Multi-Gaussian model, effectively representing the FOOOF fit of the periodic signal. The signal obtained from this fitted Multi-Gaussian is subtracted from the original signal, leading to a refitting of the aperiodic signal and yielding the final parameters for the aperiodic component.

The frequency range of the fitted FOOOF model was 0.5 to 100, and the settings for the model were a maximum of 6 peaks, peak width was 0.5 to 15, peak height was 0.10, and knee aperiodic mode was used.

After fitting the PSD using the FOOOF algorithm, the quality of the FOOOF model fit is assessed by calculating the variance (*R²*) between the original signal and the FOOOF-fitted model. We chose to exclude FOOOF-fitted models with R² values below 0.99 from the aperiodic component analyses, as these could distort the results significantly. The aperiodic signals, derived from the component parameters, were subtracted from the original signal, allowing us to isolate the periodic signal for analysis. The results of the aperiodic signal were used in the analyses, and periodograms were generated in the same manner as in the previous visualizations of the PSD values.

## Supplementary Information

Below is the link to the electronic supplementary material.

Supplementary Material 1

## Data Availability

The EEG data collected from the subjects in this study is classified as confidential and, therefore, cannot be shared publicly. However, we are open to granting access upon request. To obtain permission, please contact us with a brief explanation of your intended use, ensuring that it complies with the European Union’s GDPR (General Data Protection Regulation) regulations. For access to Dataset 1, please contact Prof. Anu-Katriina Pesonen. For Dataset 2, kindly contact Prof. Eus J. W. Van Someren.The codes and algorithms utilized in this study are available for free under the MIT license on Gitlab (https://version.helsinki.fi/sleep-mind/eegsleepmind.git).
