# Supplementary material for: Trait-like individual signatures dominate sleep EEG over insomnia-specific features
Source: Sci Rep. 2026 Jan 6;16:4408. doi: 10.1038/s41598-025-34509-y (PMC12864933; doi:10.1038/s41598-025-34509-y)
Supplement: Supplementary file 1 — Supplementary Material 1 [file 41598_2025_34509_MOESM1_ESM.docx]

Supplementary Material

# Channel artifact detection

**Fig. 1:** *Channel artifact appearance in polysomnography.*


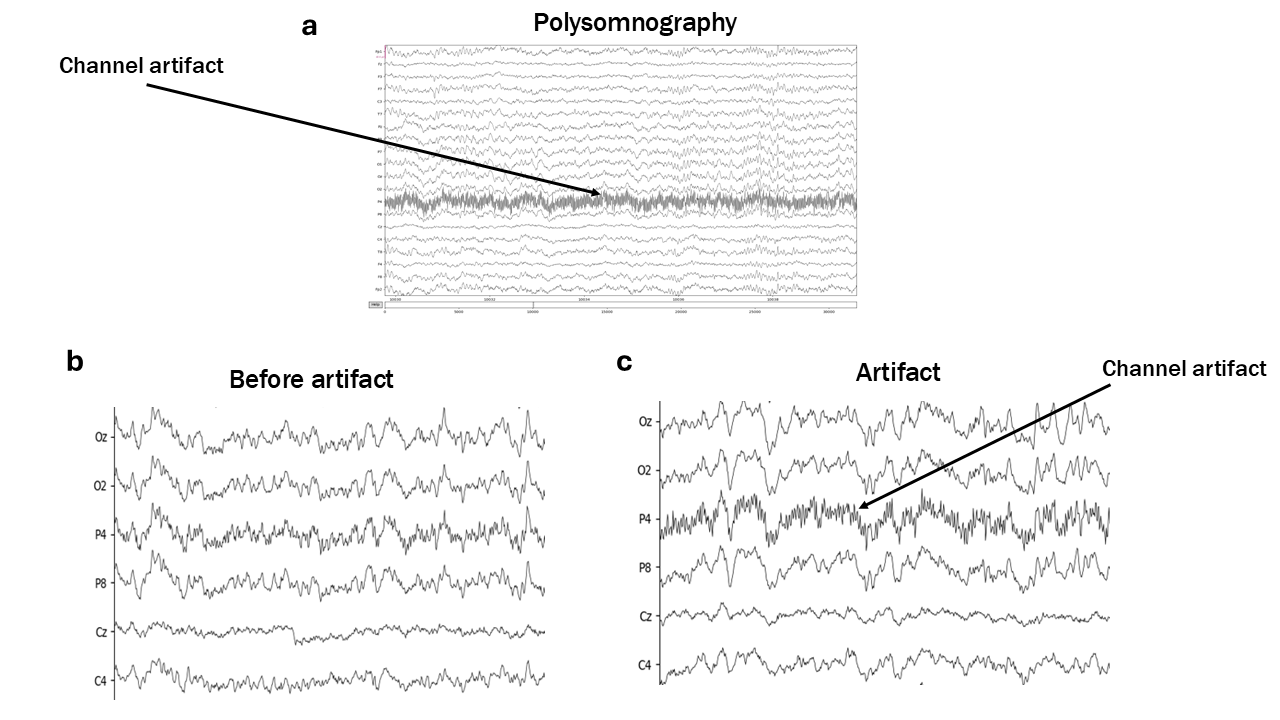


**Fig. 1 legend.** *Polysomnography demonstrates a distinct sawtooth pattern in the EEG channel signal, which is recognized as a channel artifact (a). Panel (b) illustrates the polysomnography reading before the emergence of the channel artifact, while panel (c) shows the reading after its formation.*

# Linear methods

**Fig. 2:** *The boxplot indicates no differences in insomnia severity related to brain wave activity across datasets and in REM and NREM sleep stages.*


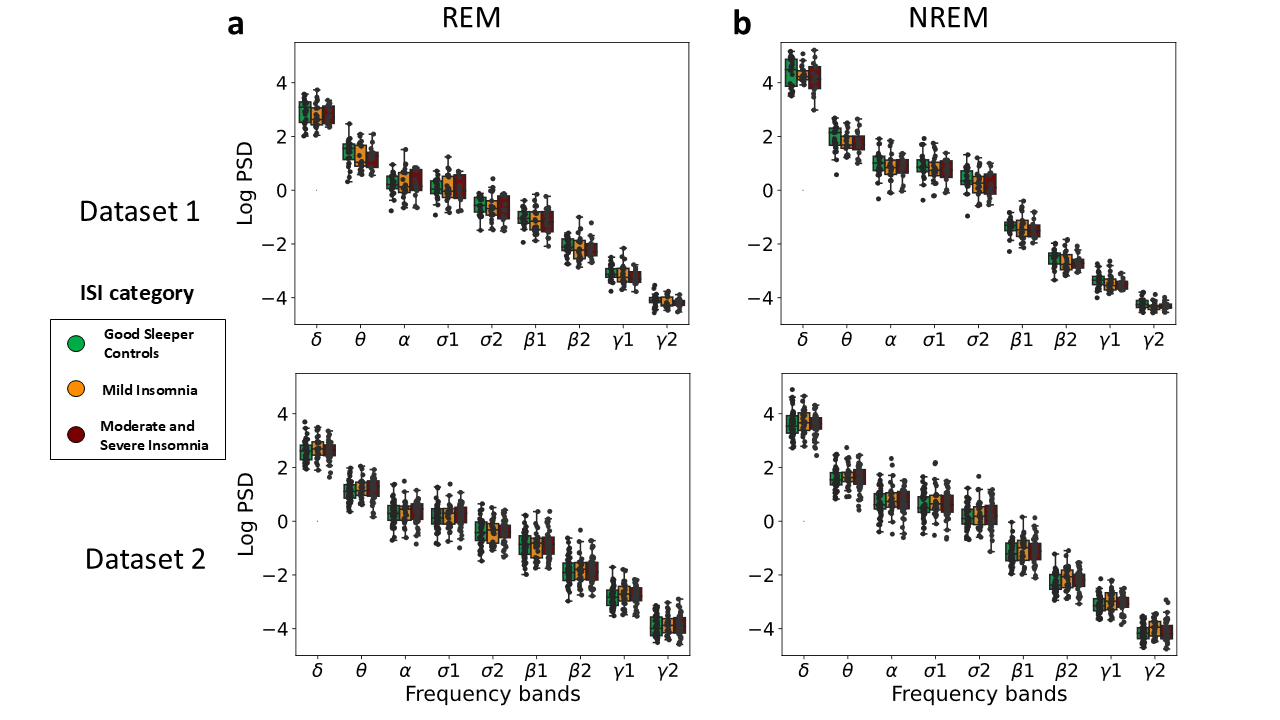
**Fig. 2 legend.** *Boxplots show the absolute log power spectral density (PSD) values of each brain activity in REM (a) and NREM (b) sleep with insomnia severity group comparison. The black dots illustrate the subjects’ average PSD value for the corresponding frequency bands. δ = delta (0.5-3Hz); θ = theta (4-7 Hz); α = alpha (8-12.5 Hz); σ1 = low sigma (9-12.5 Hz), σ2 = high sigma (12.5-16 Hz); β1 = low beta (16-24 Hz); β2 = high beta (25-35 Hz); γ1 = low gamma (35-48 Hz); γ2 = high gamma (52-100 Hz).*

**Fig. 3:** *Boxplots reveal that adjusting for sex and age did not change the results for relative and absolute PSD.
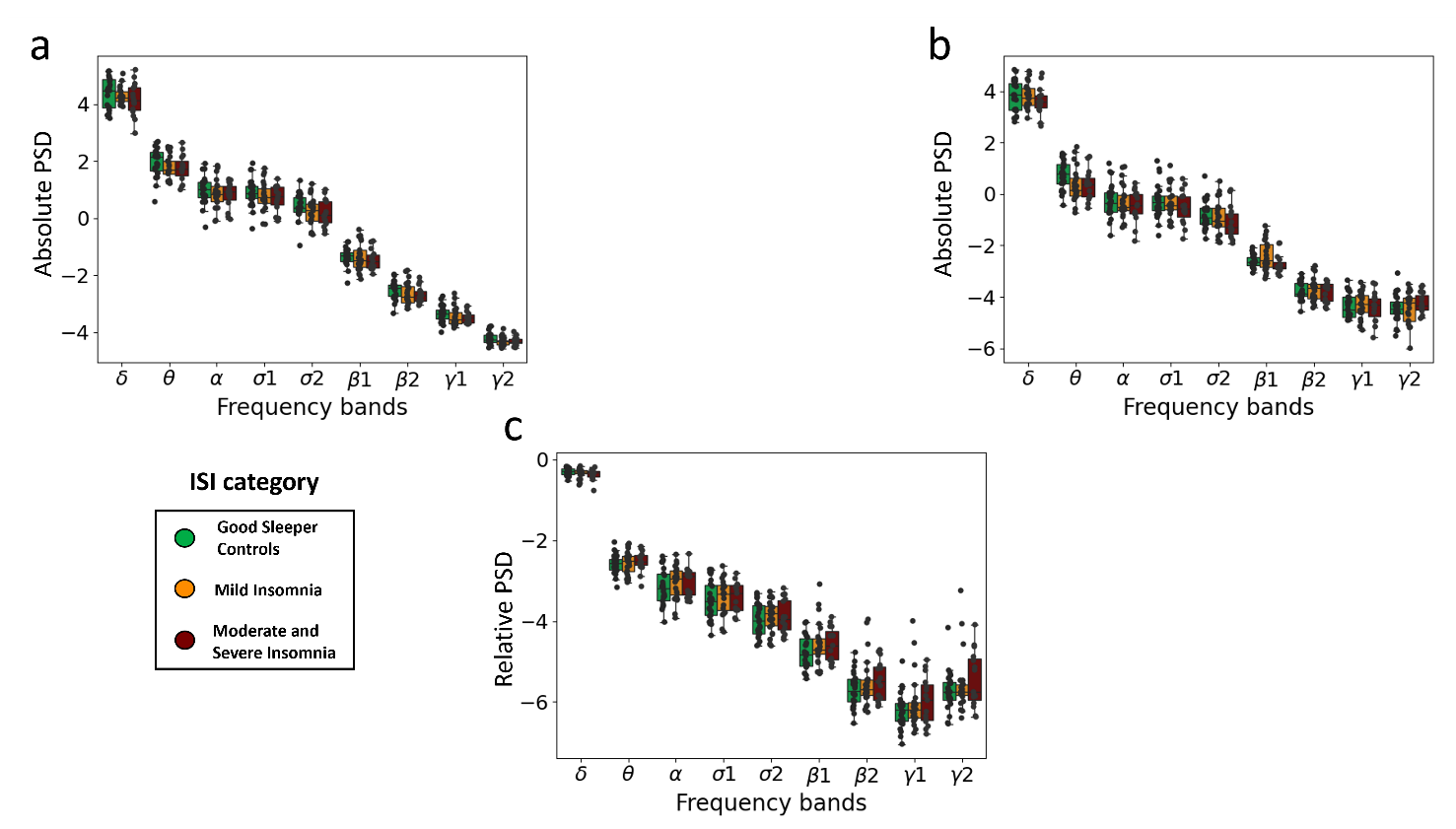
***Fig. 3 legend.** *Boxplots illustrate the natural log transformation of the PSD (a), the Absolute PSD adjusted for age and sex (b), and the relative PSD (c) of each brain activities during NREM sleep from Dataset 1. The analysis takes into consideration comparisons among different insomnia severity groups. The black dots represent the average PSD values for participants across the respective frequency bands.*

**Fig. 4:**  *Correlation between Insomnia Severity Index (ISI) values and age in the datasets.*


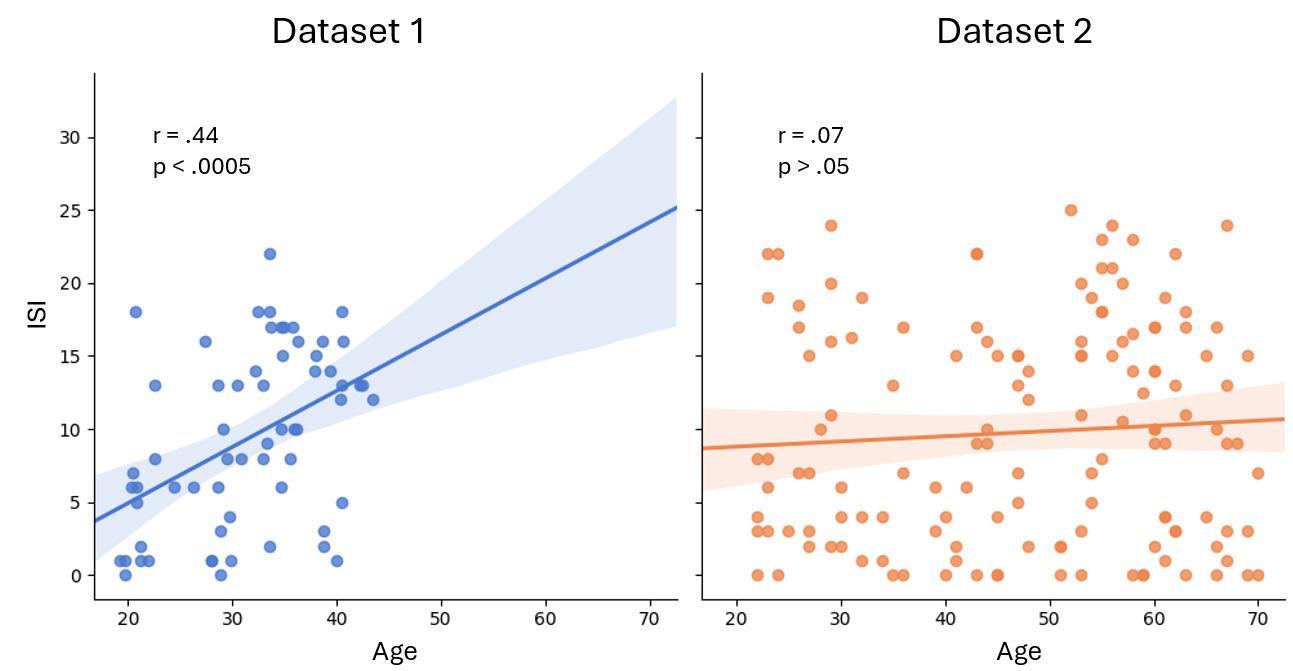
**Fig. 4 legend.** *Scatterplots illustrate the correlation between Insomnia Severity Index (ISI) values and age across two datasets. Dataset 1 is displayed on the left, while Dataset 2 is on the right. Each independent sample represents an individual subject. The dark lines indicate linear regression, and the shaded area around these lines represents the 95% confidence interval.*

# **Supervised learning classifier**

To evaluate the performance of the classification models, we used four different metrics: accuracy, precision, recall, and F1 score. Accuracy was calculated as the overall performance using the following equation (2):

| $\text{Accuracy}=\frac{TP+TN}{TP+FP+TN+FN},$ | (2) |
| --- | --- |

where TP, FP, TN and FN are true positives, false positives, true negatives and false negatives of test data respectively.

Precision, often referred to as positive predictive value, measures the accuracy of the positive predictions made by a model, highlighting any misclassifications. In contrast, recall evaluates the model's effectiveness in identifying relevant patterns and is also known as sensitivity. The F1 score captures the harmonic mean of precision and recall, serving as an overall performance metric. These metrics can be defined by the following formulas:

| $\text{Precision}=\frac{TP}{TP+FP}$ | (3) |
| --- | --- |
| $\text{Recall}=\frac{TP}{TP + FN}$ | (4) |
| $F1=2\cdot\frac{\text{Precision}\cdot\text{Recall}}{\text{Precision}+\text{Recall}}$ | (5) |

**Fig. 5:** *Confusion matrices for XGBoost classifiers across various datasets and in REM and NREM sleep stages.*


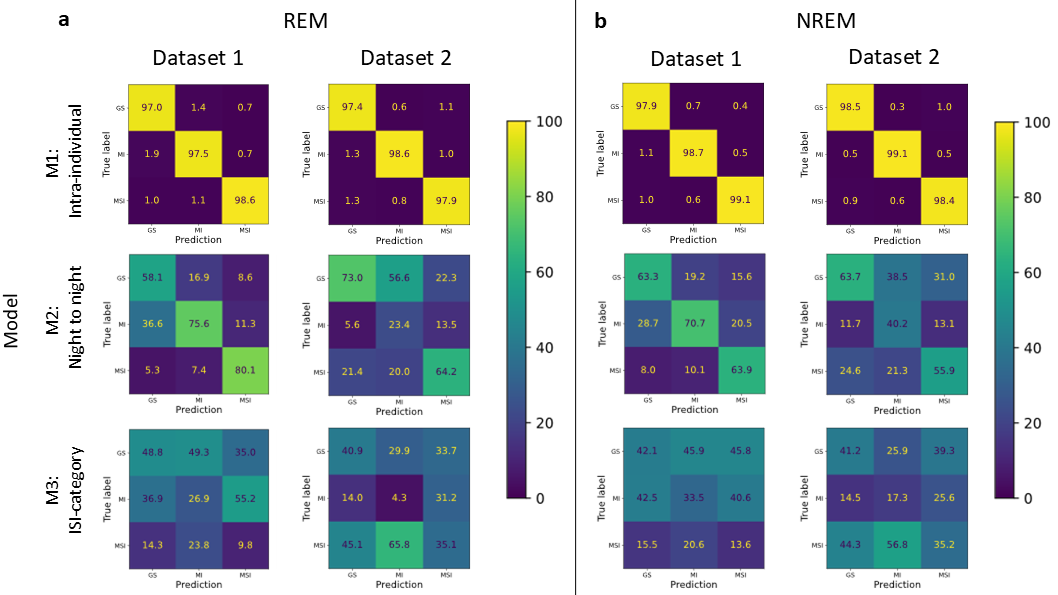


**Fig. 5 legend.** *The confusion matrices display the performance of the XGBoost classifier, which is used to categorize insomnia severity groups based on brain wave activities recorded during sleep epochs in REM (a) and NREM (b) sleep across datasets. Each row represents an instance of an actual label, and each column represents an instance of model prediction for a specific classification group. Yellow indicates a high percentage of correct classifications, dark blue signifies a low percentage, and green represents a medium percentage of classification, roughly 50%.*

# Unsupervised learning model

t-SNE, a nonlinear dimension reduction technique developed by van der Maaten et al., employs pairwise joint probabilities to analyze distances between data points. The method considers pairwise data points in both high-dimensional and low-dimensional spaces. It calculates the probabilities of the distances between these pairs using the normalized Student's t-distribution. The Euclidean distance is commonly used as the distance metric in this context. The following formula is used to determine the probability distribution in the high-dimensional space (p) with t-SNE:

| $p_{j\vert i}=\frac{exp(-\left\Vert x_{i}-x_{j} \right\Vert^{2}/2{\sigma_{i}}^{2})}{\sum_{k\neq i} exp(-\left\Vert x_{i}-x_{k} \right\Vert^{2}/2{\sigma_{i}}^{2})},$ | (6) |
| --- | --- |

where $x_{N}$ represents a set of *N* high-dimensional data points and $\sigma_{N}$ represents a set of *N* standard deviations. The value of $p_{ij}$ is calculated using the probabilities $p_{j|i}$ with the formula:

| $p_{j\vert i}=\frac{p_{j\vert i}+p_{i\vert j}}{2N}$ | (7) |
| --- | --- |

This was also performed for the low-dimensional space, where low-dimensional probability distribution (q) is calculated using the following equation:

| $q_{ij}=\frac{\left( 1+\left\Vert y_{i}-y_{j} \right\Vert^{-1} \right)}{\sum_{k} \sum_{l\neq k} \left( 1+\left\Vert y_{k}-y_{l} \right\Vert^{2} \right)^{-1}},$ | (8) |
| --- | --- |

where $y_{N}$ is a set of *N* low-dimensional data points. To effectively align the low-dimensional probability distribution with the high-dimensional probability distribution, the t-SNE algorithm uses the Kullback-Leibler (KL) divergence as its loss function. This divergence is calculated using the formula:

| $KL\left( P\vert\vert Q \right)=\sum_{i\neq j} p_{j\vert i}\log\frac{p_{ij}}{q_{ij}},$ | (9) |
| --- | --- |

where probability distribution *P* is high-dimensional space, and *Q* is low-dimensional space. The *Q* distribution is adjusted to closely match the *P* distribution. Perplexity is defined by the equation:

| $\text{Perp}\left( P_{i} \right)=2^{H\left( P_{i} \right)},$ | (10) |
| --- | --- |

where $H\left( P_{i} \right)$ is the Shannon entropy of $P_{i}$ in pits. The Shannon entropy of $P_{i}$ can be calculated using the formula:

| $H\left( P_{i} \right)=-\sum_{j} p_{j\vert i}\log_{2} p_{j\vert i}$ | (11) |
| --- | --- |

Typically, the value of the perplexity parameter ranges from 5 to 50 to get the data into a representative format.

**Fig. 6:** *PCA visualization of Dataset 2 during NREM sleep: original and zoomed graphs without outliers.*


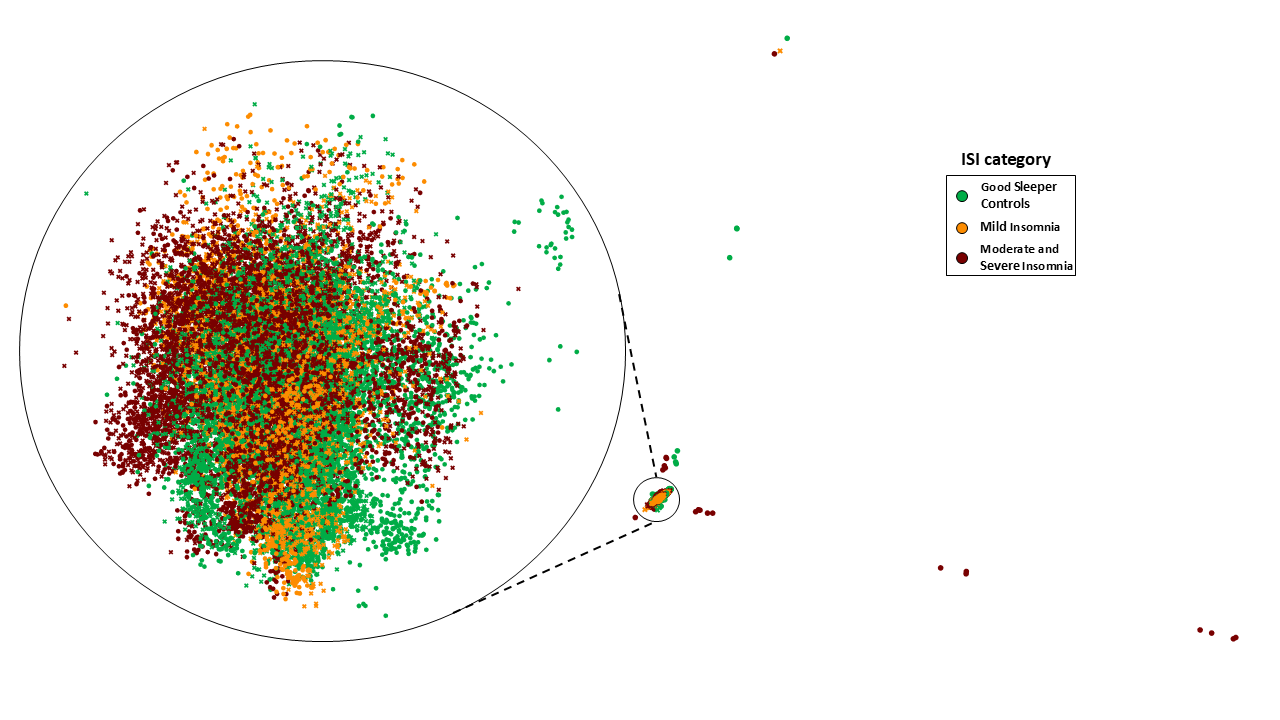


**Fig. 6 legend.** *The Principal Component Analysis* (*PCA) visualization of Dataset 2 illustrates the 2D locations of brain wave activity during NREM sleep epochs with independent samples categorized by the severe insomnia group. The circled frame highlights the magnified area of the plot.*

**Fig. 7:** *Dimension reduction methods did not reveal any exceptions in standardization, or EMG activity during REM sleep in Dataset 1.*

*
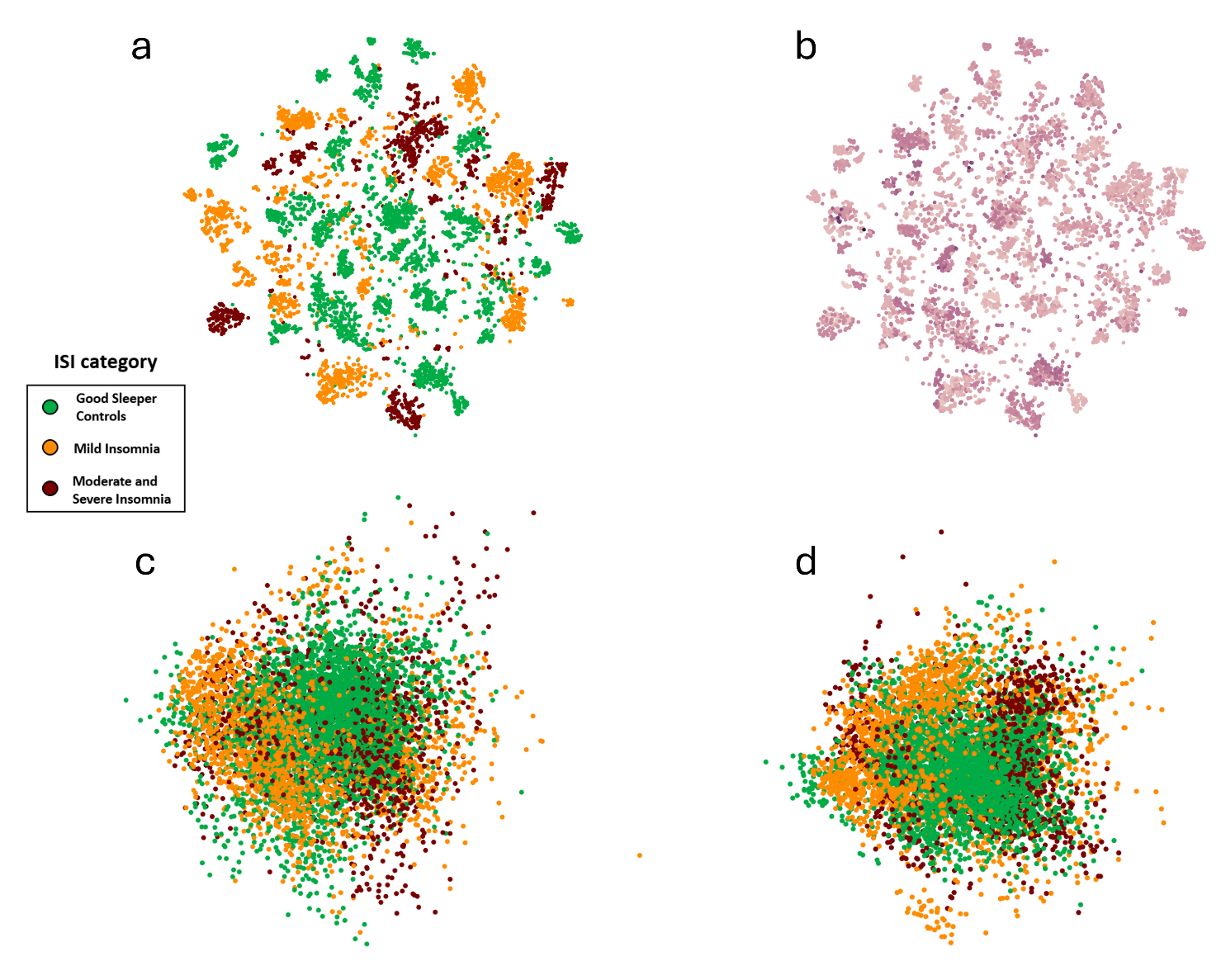
*

**Fig. 7 legend.** *The t-SNE plots show the absolute log PSD values for the insomnia severity index (ISI) (a) and electromyography (EMG) root mean square (RMS) (b) from 20 EEG channels across nine frequency bands during 30-second REM sleep epochs. The PCA plots present the same absolute log PSD values as the t-SNE plots, but with log normalization (c) or standardization (d).* *Lighter colours indicate lower RMS values, while darker colours indicate higher RMS values.*
